# Understanding the Impact of Chain Mobility on Conformational Evolution and Kinetics of Mesophase Formation in Poly(ʟ-lactide) under Low-Pressure CO_2_

**DOI:** 10.3390/polym16101378

**Published:** 2024-05-12

**Authors:** Youjuan Liao, Qiaofeng Lan

**Affiliations:** Biomaterials Research Center, School of Biomedical Engineering, Southern Medical University, Guangzhou 510515, China; liaoyjcn@126.com

**Keywords:** polylactide, mobility, mesophase, carbon dioxide, structural relaxation, dynamics

## Abstract

Although the mesomorphic phase as an intermediate state has been introduced to understand polymer crystallization, the understanding of the mesomorphic phase is far from complete. Here, the effect of chain mobility on the mesophase structuring in melt-quenched poly(ʟ-lactide) (PLLA) treated in low-pressure CO_2_ at 1.6–2.0 MPa and 0 °C was investigated using infrared (IR) spectroscopy, differential scanning calorimetry (DSC), and atomic force microscopy (AFM). The IR and AFM results demonstrated that the final degree of order and the kinetics of structural evolution during the CO_2_-induced mesophase formation were critically dependent on the CO_2_ pressure. This was attributed to the distinct dynamics of conformational evolution (*gg* to *gt* conformer transition) due to the different CO_2_ pressures. The thermal behavior from the DSC results showed that CO_2_ pressure dominated both the scale and dynamics of the chain motion of PLLA. At a lower CO_2_ pressure of 1.6 MPa, smaller-scale segmental motion was not replaced by the larger-scale cooperative motion that occurred at a relatively higher CO_2_ pressure of 2 MPa, which was favorable for faster mesophase formation. Consequently, by inhibiting direct crystallization under limited mobility conditions, it was demonstrated that different chain mobility controlled by CO_2_ pressure and thus CO_2_ solubility impacted the dynamics of the mesophase formation of PLLA. The present results have implications for understanding the role of chain mobility in determining the intermediate structural phases in semicrystalline polymers.

## 1. Introduction

Because of its high biocompatibility, good processability, and considerable mechanical properties, poly(ʟ-lactide) (PLLA) exhibits wide applications in various fields such as packaging materials, medical devices, and textiles [1,2,3,4,5,6,7,8]. As a semicrystalline polymer, the physical properties of PLLA can be regulated by controlling its crystalline structure and morphology [8,9,10,11,12,13]. Specifically, by controlling the processing or crystallization conditions, the crystal orientation, crystallinity, and chain packing modes of PLLA can be tailored. For instance, as a polymorphic polymer, PLLA can crystallize into distinct crystal modifications including α, α′(δ), α″, β, γ, ε, and stereocomplex (with its enantiomer) crystals [12,13,14,15,16]. In addition, as an intermediate ordered structure having an order between the amorphous and crystalline states, a mesomorphic phase (mesophase) can also be formed under specific conditions in PLLA [17,18,19,20,21].

The intermediate phase and other moderately ordered structures are of great significance for understanding the crystallization mechanism of polymers. Differing from the HL (Hoffman and Lauritzen) model of crystal growth [22,23,24], it is proposed that the formation and growth of lamellar crystals in polymer crystallization is a multistep process passing through an intermediate phase (mesomorphic phase) [25,26,27], which exists as a pre-ordered phase with intermediate state characteristics at the front during crystal growth. On the other hand, the mesomorphic phase can be utilized to control the crystallization and structural morphology of polymers including PLLA [28,29,30,31,32]. Taking isotactic polypropylene (iPP), its copolymers, and polyamides as examples, the size of the resulting crystalline entity is very small, typically on the nanoscale, after crystallization through the pathway via the mesophase [30,31,32]. Similarly, the mesophase-mediated crystallization of PLLA results in a nonspherical crystalline morphology, which is attributed to the role of nuclei from the mesophase [28]. In homopolymers, a very limited amount of mesophase can be induced in melt-quenched PLLA by prolonged physical aging at temperatures near the glass transition temperature (*T*_g_) [19]. The mesophase can also be formed in stretched PLLA near its *T*_g_ [17,18]. In comparison, small molecules and low-molecular-weight polymers, e.g., poly(ethylene glycol) (PEG), with a low *T*_g_ can act as blending components to induce PLLA to form a mesophase at temperatures below the *T*_g_ [33,34]. In addition, the incorporation of block structures with plasticizing effects into copolymers, such as the triblock copolymers PLLA-*b*-PEG-*b*-PLLA and poly(ʟ-lactide-*b*-dimethylsiloxane-*b*-ʟ-lactide) (PLLA-*b*-PDMS-*b*-PLLA), can also lead to the formation of an intermediate phase at temperatures below the *T*_g_ [35,36,37]. In the above systems, a mesophase can only be achieved at relatively low temperatures instead of high temperatures. This is because high molecular mobility at high temperatures would lead to direct crystallization. In our previous work, we investigated mesophase formation using low-pressure CO_2_ and proposed that the condition for the formation of the intermediate phase is moderately increased molecular mobility [20].

Although small molecules and low-molecular-weight polymers can enhance molecular mobility to some extent as blending components or block structures and can promote the formation of the mesophase, previous studies have only focused on individual compositions. As such, the impact of the content of plasticizing components on mesophase formation has not been discussed. Moreover, the blending components or block structures cannot be removed after mesophase formation or have to be considered with PLLA during mesophase formation, which may interfere with structural characterization. Therefore, the impact of molecular mobility on the formation of the mesophase is still unclear. In contrast, as a gaseous plasticizer, CO_2_ can be quickly and completely removed from the polymer matrix. More importantly, PLLA can exhibit varying molecular mobility by simply adjusting the pressure and temperature.

In this work, we investigate the impact of varying molecular mobility on mesophase formation by adjusting CO_2_ pressures within the low-pressure range at the low temperature of 0 °C. The kinetics of the mesophase and morphological changes under various CO_2_ pressure conditions are monitored using infrared spectroscopy and atomic force microscopy (AFM), respectively. Differential scanning calorimetry (DSC) is used to study the thermal behavior with varying degrees of intermediate phase ordering to elucidate the scale of chain motion during mesophase formation. This work aims to gain insights into the relationship between chain mobility (and scale of motion) and conformational evolution during mesophase formation in PLLA.

## 2. Materials and Methods

### 2.1. Materials and Sample Preparation

Poly(ʟ-lactide) (PLLA) (grade PURASORB PL18), with an inherent viscosity and weight-averaged molecular weight of 1.61 dL/g and 1.8 × 10^5^ g/mol, respectively, was kindly supplied by Corbion Purac (Amsterdam, the Netherlands). CO_2_ with a purity of 99.99% was supplied by Air Liquide Co., Ltd., Foshan, China. Dichloromethane (CH_2_Cl_2_) (AR grade) was used as received. A PLLA film for the CO_2_ treatment was cast onto a clean substrate (ca. 130 μm thick cover glass) from CH_2_Cl_2_ solution (10 mg/mL of PLLA). After the majority of the solvent had evaporated, the film was placed under vacuum at room temperature for 24 h and then at 50 °C for 48 h to completely remove the residual solvent. The prepared PLLA film had a thickness ca. 5 μm. Note that such a thickness permits avoiding the possible effect of the sorption or desorption process of CO_2_ on the structural change (e.g., foaming) in the film sample. The film was quickly quenched to 0 °C within 1 s after melting at 210 °C for 30 s to erase any thermal history and obtain the glassy sample.

### 2.2. CO_2_ Treatment

Since high-pressure CO_2_ at higher temperatures certainly induces crystallization [38,39,40,41,42], in this work, all CO_2_ treatments for the glassy PLLA films were conducted at low pressures of 1.6–2.0 MPa. The glassy PLLA films were treated by CO_2_ at a fixed temperature of 0 °C for a certain treatment time (*t*_t_) in a high-pressure vessel connected to a liquid-CO_2_-filled high-pressure cylinder. Before the pressurization for treatment, the vessel was flushed with an extremely low-pressure CO_2_ flow for about 8 min. After treatment, the vessel was depressurized steadily at an approximate rate of 2 MPa/min. The treated samples were kept under dry atmosphere for at least two weeks prior to measurements.

### 2.3. Characterization

The Fourier transform infrared (FTIR) spectroscopic analysis of untreated PLLA or treated PLLA was performed on a Thermo-Nicolet IS50 FTIR spectrometer. Transmission scans were conducted between wavenumber of 4000 and 400 cm^−1^ at a resolution of 2 cm^−1^ and scan numbers at 64. All of the spectra were baseline-corrected and then normalized at fixed wavenumbers around 1350 and 867 cm^−1^ for wavenumber ranges of 1850–1080 and 970–850 cm^−1^, respectively. A TA Instruments DSC250 differential scanning calorimeter (DSC) was used for standard DSC analysis. All measurements were carried out with ca. 3 mg samples from 0 to 200 °C under a nitrogen flow of 50 mL/min. A heating rate of 10 °C/min was used. Before FTIR and DSC measurements, free-standing PLLA films were obtained by floating the samples off the glass substrates onto the surface of water. The films were then allowed to dry thoroughly in vacuum at room temperature prior to measurements.

The nanoscopic surface morphology and roughness of the film samples on substrates of cover glass were obtained at room temperature using an atomic force microscope (AFM) (Dimension FastScan, Bruker Nano Inc, Santa Barbara CA, USA) operating in tapping mode. Both height and peak force images were recorded simultaneously using the retrace signal. Silicon tips with a resonance frequency of 450 kHz and a spring constant number of 1.8 N/m were used, and the scan rate was 3.76 Hz with the scanning density of 256 lines/frame.

## 3. Results and Discussion

**Figure 1:**
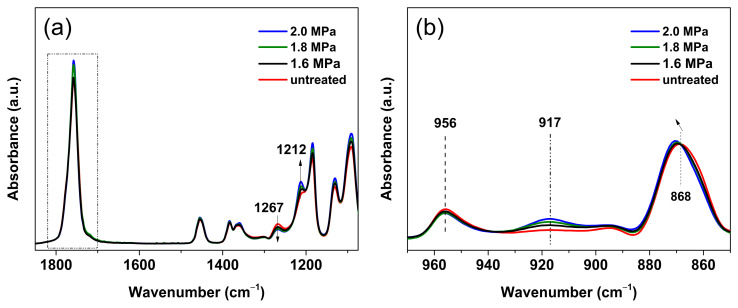
FTIR spectra in the wavenumber ranges of (**a**) 1850–1080 and (**b**) 970–850 cm^−1^ for PLLA films treated under CO_2_ at 1.6–2.0 MPa and 0 °C for treatment time (*t*_t_) of 12 h. The spectrum of melt-quenched/untreated sample is also shown for comparison.

We first investigated the structural order and conformational evolution of melt-quenched PLLA film samples previously treated in low-pressure CO_2_ using FTIR spectroscopy. Figure 1 (for the full range of wavenumbers, see Figure S1) shows the FTIR spectra in the wavenumber range of 1850–850 cm^−1^ for PLLA treated with different pressures (1.6–2.0 MPa) of CO_2_ at 0 °C for a sufficiently long period of *t*_t_ = 12 h. The bands in the range of 1500–1000 cm^−1^ (Figure 1a) are mainly attributed to C–H, CH_3_ bending, and C–O–C stretching, while the characteristic peaks of C–C skeletal stretching coupled with CH_3_ rocking are mainly located at 970–850 cm^−1^ (Figure 1b) [43]. Compared with the melt-quenched sample, the IR spectra undergo significant changes upon CO_2_ treatment, particularly in the lower wavenumber range. The intensity of the 1267 cm^−1^ band arising from the amorphous phase decreases significantly, while the intensity of the 1212 cm^−1^ band increases, indicating that the amorphous phase was consumed to enhance the conformational order. In addition, the 868 cm^−1^ band slightly shifts to a higher frequency and becomes sharper, confirming the formation of structural order. Moreover, it is worth noting that the intensity of the characteristic peak at 917 cm^−1^ increases. It has been reported that the characteristic peak around 915–918 cm^−1^ is attributed to the characteristic peak of the intermediate ordered phase (referred to as the mesophase) of pure PLLA or PLLA in blended or copolymer systems [20,33,34,35,36]. From the results of Figure 1b, the characteristic peak at about 921 cm^−1^ originating from the crystalline phase (α or α′ form) of PLLA is absent, suggesting that no crystalline structure was formed in these samples. Accordingly, the consumption of the amorphous phase mentioned above was due to the mesophase formation. Furthermore, it can be seen that the changes in the aforementioned characteristic peaks are clearly dependent on the CO_2_ pressure. With increasing CO_2_ pressure, the intensity of the characteristic peaks at 917 and 1212 cm^−1^ increases, while the intensity of the peak at 1267 cm^−1^ decreases. Therefore, it is demonstrated that the final degree of order of the CO_2_-induced mesophase formation is critically linked to the CO_2_ pressure.

**Figure 2:**
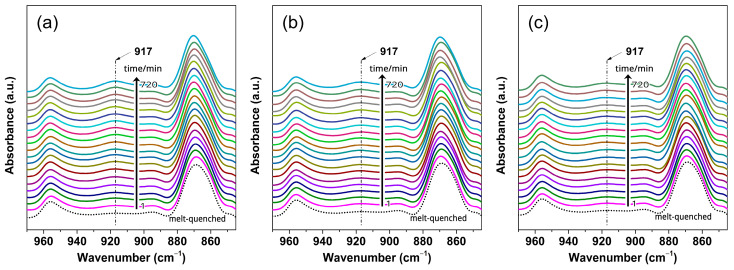
FTIR spectra in the wavenumber range of 970–850 cm^−1^ for PLLA films treated under CO_2_ at different pressures and 0 °C for different treatment times (*t*_t_): (**a**) 2.0, (**b**) 1.8, and (**c**) 1.6 MPa.

**Figure 3:**
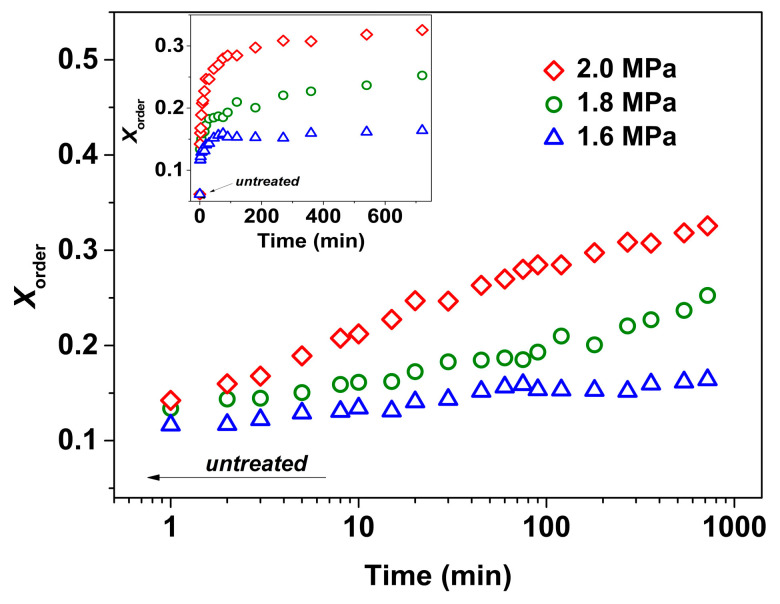
Evolution of mesophase fraction (*X*_order_) (calculated from the spectra in Figure 2) as a function of treatment times (*t*_t_) for PLLA films treated under CO_2_ at different pressures and 0 °C. The inset shows the *X*_order_ versus the linear *t*_t_. Integrated area of *A*_917_ and *A*_956_ were calculated based on baselines of 932–905 and 967–938 cm^−1^ around the 917 and 956 cm^−1^ bands, respectively.

To gain a better understanding of how CO_2_ pressure affects structural formation and evolution, ex situ FTIR measurements were further conducted. The corresponding spectra obtained from treatments under three pressures of 1.6, 1.8, and 2.0 MPa are shown in Figure 2. All PLLA samples exhibit characteristic peaks corresponding to the mesophase, with their intensity increasing with increasing *t*_t_, indicating the formation and evolution of the mesophase. The degree of structural order (*X*_order_) for the mesophase was analyzed to quantitatively compare the evolution process of order during the mesophase formation. The *X*_order_ calculation was performed according to the equation *X*_order_ = *A*_917_/(*A*_917_ + *A*_956_), where *A*_917_ and *A*_956_ are the integrated areas of the 917 and 956 cm^−1^ bands, respectively; the results are shown in Figure 3. The *X*_order_ of the melt-quenched sample is about 6%. Upon treatment, *X*_order_ increases at a faster rate in the shorter *t*_t_ range and then slows down at the later stage. When compared to the untreated sample, the final *X*_order_ enhancements are approximately 10, 19, and 27% for treatments at 1.6, 1.8, and 2.0 MPa, respectively. Therefore, it is demonstrated that the kinetics of structural evolution during the mesophase formation depend on the CO_2_ pressure.

**Figure 4:**
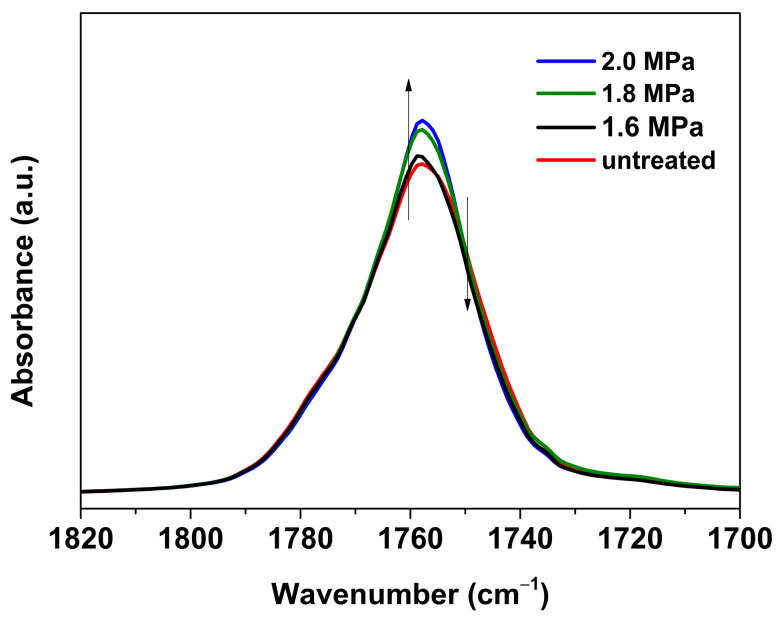
FTIR spectra in the wavenumber ranges of 1820–1700 cm^−1^ for PLLA films treated under CO_2_ at 1.6–2.0 MPa and 0 °C for treatment time (*t*_t_) of 12 h. The spectrum of melt-quenched/untreated sample is also shown for comparison.

**Figure 5:**
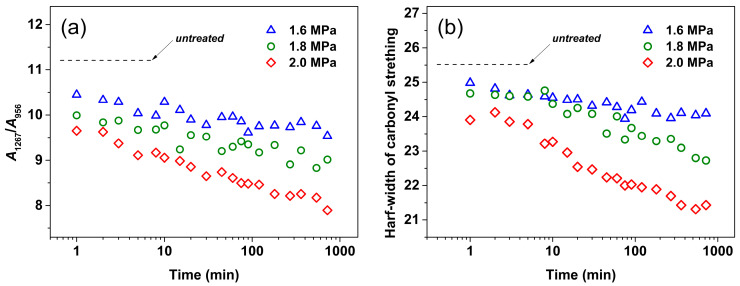
(**a**) Intensity ratio *A*_1267_/*A*_956_ and (**b**) evolution of half-width of the carbonyl stretching region (1820–1700 cm^−1^) as a function of treatment time (*t*_t_) for PLLA films treated under CO_2_ at different pressures and 0 °C. Integrated area of *A*_1267_ and *A*_956_ were calculated based on baselines of 1287–1252 and 967–938 cm^−1^ around the 1267 and 956 cm^−1^ bands, respectively.

In addition, the characteristic band located in the 1800–1700 cm^−1^ range is attributed to the carbonyl stretching mode, which can provide information on the energetically favorable *gt* conformations. As shown in Figure 4, upon mesophase formation, the width of the characteristic band is slightly narrower compared to that of the untreated sample. Combined with the evolution of the characteristic peak at 1267 cm^−1^ (reflecting the conformational population of higher energy *gg*) [44], the CO_2_ pressure dependence of the conformational evolution during the mesophase formation can be discussed. Figure 5a shows the trend plot of the intensity ratio of *A*_1267_/*A*_956_, in which *A*_1267_ and *A*_956_ are the integrated areas of the 1267 (*gg* conformer sensitive) and 956 cm^−1^ bands, respectively. It can be seen that the *A*_1267_/*A*_956_ ratio of samples under each pressure declines with increasing *t*_t_ as compared with the melt-quenched sample, indicating that a reduction in the population of the less energetically favorable *gg* conformers accompanied the mesophase formation. Additionally, a higher CO_2_ pressure results in a more pronounced and faster reduction in the *A*_1267_/*A*_956_ ratio, which demonstrates that the rate of structural evolution and *X*_order_ during mesophase formation are strongly dependent on the decrease in population of the *gg* conformers. On the other hand, as shown in Figure 5b, the narrowing trend of the half-width of the carbonyl stretching vibrational region (which is energetically favorable and sensitive to the *gt* conformation [45,46]) of PLLA samples under different pressures is essentially similar to the case of the *A*_1267_/*A*_956_ ratio, indicating that the increase in *gt* conformers occurred at the expense of the decrease in *gg* conformer population. Thus, the evolution of energetically favorable *gt* conformers (narrowing of half-width) also shows CO_2_ pressure dependence.

It is noteworthy that the difference (<ca. 4) (Figure 5b) in half-width before and after mesophase formation is much smaller than that during crystallization [45], even in the case of the maximum pressure of 2 MPa. Furthermore, no crystalline order (corresponding to perfect helices) was formed after CO_2_ treatment under 1.6–2.0 MPa. Thus, it can be argued that the formed mesophase exhibits an intermediate degree of conformational order between crystalline phase and amorphous state with random coils. In particular, for the situation of the lowest pressure (1.6 MPa), only a very limited *gg*-*gt* conformational transition occurred even after a sufficiently long *t*_t_; as such, the degree of order of the mesophase under 1.6 MPa is still very low. Therefore, it is concluded that CO_2_ pressure determines the dynamics of conformational evolution (*gg* to *gt* conformer transition), resulting in varying degrees of order and evolution kinetics in mesophase formation.

**Figure 6:**
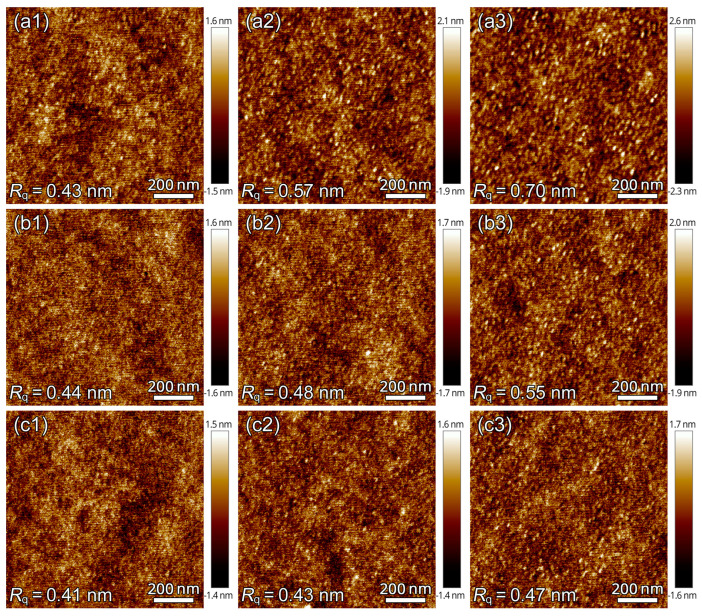
AFM height images for PLLA films treated under CO_2_ at different pressures of (**a1**–**a3**) 2.0, (**b1**–**b3**) 1.8, and (**c1**–**c3**) 1.6 MPa and 0 °C for different treatment times (*t*_t_) of (**a1**–**c1**) 3, (**a2**–**c2**) 30, and (**a3**–**c3**) 300 min.

Figure 6 shows the representative AFM nanostructured morphology of PLLA samples treated with different CO_2_ pressures. For the relatively long time period of *t*_t_ = 300 min, PLLA film samples CO_2_-treated at three pressures were filled with numerous nanoscale granules with sizes of about 18–25 nm. That is, the entire sample can be considered to be divided into nanoscale domains. Thus, in combination with the aforementioned IR results, the mesophase formation under different CO_2_ conditions actually corresponds to the structural evolution in the nanoscale granules. For the same *t*_t_, with increasing CO_2_ pressure, the morphological appearance of nanoscale granules becomes more pronounced. For instance, at 2 MPa, the sample of *t*_t_ = 30 min shows clear nanoscale granules. As the treatment pressure decreases, the formation and evolution of nanoscale granules slow down. For the lower CO_2_ pressure conditions of 1.8 and 1.6 MPa, almost no clear nanoscale granules can be found after a shorter *t*_t_ = 3 min. In terms of root-mean-square roughness (*R*_q_), the fastest change in *R*_q_ is observed for high pressure, while the change in *R*_q_ is slower for lower pressure. Therefore, the AFM morphology results also further support that the CO_2_ treatment pressure affects the formation process of the mesophase.

**Figure 7:**
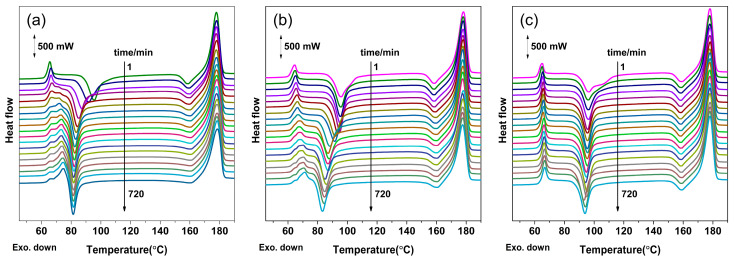
DSC curves for PLLA films treated under CO_2_ at different pressures and 0 °C for different treatment times (*t*_t_): (**a**) 2.0, (**b**) 1.8, and (**c**) 1.6 MPa.

As seen in Figure 6, because of the ultra-high nucleation density of the structural ordering, the sample is considered to be approximately divided into numerous nanoscale regions. The sample can be composed of granular intermediate ordered structures (mesophase) with varying degrees of order, which is strongly dependent on the *t*_t_. Thus, the degree of order and structural evolution of the mesophase during the formation process under various treatment conditions (i.e., *t*_t_ and pressure) can be further understood by studying the ensemble thermal behavior. As shown in Figure 7, the DSC heating curves exhibit multiple thermal transition signals, in particular the enthalpy recovery, endothermic peak (*T*_g, post_) near the glass transition, and cold crystallization corresponding to the peak temperature of *T*_c_, depending on the treatment conditions.

**Figure 8:**
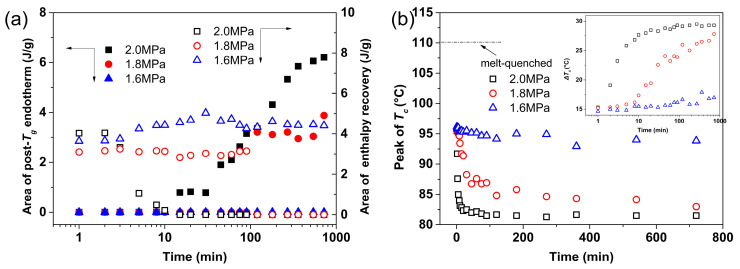
(**a**) Peak areas of enthalpy recovery peak and post-*T*_g_ endotherms and (**b**) peak temperatures of cold crystallization (*T*_c_) for PLLA films treated under CO_2_ at different pressures and 0 °C for different treatment times (*t*_t_). The data were derived from the DSC curves shown in Figure 6.

Before CO_2_ treatment, the melt-quenched PLLA sample underwent enthalpy relaxation [47], which is a process of evolving towards equilibrium by reducing the excess in its thermodynamic properties [48], such as enthalpy. Owing to previous enthalpy relaxation, the glass transition overlaps with an endothermic peak known as enthalpy recovery during heating in DSC. As shown in Figure 7 and Figure 8a, after CO_2_ treatment, the enthalpy recovery peak weakens and even disappears with increasing *t*_t_. In particular, for the samples at 2 MPa, the enthalpies of the enthalpy recovery peak start to decrease even at a very short *t*_t_ of 3 min and decay to zero at about *t*_t_ = 10 min. Simultaneously, the *T*_g, post_ peak, which is proposed to be associated with the mesophase, appears and its area increases with increasing *t*_t_. Enthalpy recovery can be considered as the reverse process of enthalpy relaxation, which is commonly linked to smaller-scale dynamics compared to the larger-scale cooperative motion associated with *T*_g_ [48]. Thus, the concurrent decrease/disappearance of enthalpy recovery and the appearance/enhancement of the *T*_g, post_ peak demonstrate that the enthalpy relaxation with smaller-scale motion had been replaced by cooperative segmental motion on a much larger scale. This transition occurs at about 10^1^ min and accounts for the faster mesophase formation at 2 MPa. For the case of 1.8 MPa, this transition occurs at a longer *t*_t_ of about 10^2^ min although the enthalpy recovery and *T*_g, post_ peaks actually overlap. In contrast, all samples treated at 1.6 MPa only exhibit enthalpy recovery peaks without any trace of the *T*_g, post_ peak, while the area of enthalpy recovery remains almost constant, indicating that the aggregated state formed by enthalpy relaxation had not been replaced or destroyed. This result demonstrates that despite the mesophase formation at 1.6 MPa, the scale of chain motion is much smaller than those at 1.8 and 2.0 MPa. Therefore, it can be concluded that CO_2_ pressure affects the scale of chain motion during mesophase formation.

In addition, as shown in Figure 7a,b and Figure 8b, the *T*_c_ of the samples treated under higher CO_2_ pressures (1.8 and 2.0 MPa) significantly decreases with increasing *t*_t_, indicating highly enhanced crystallization kinetics compared to the melt-quenched sample (Figure S2). As an intermediate structural order, the mesophase can transform into a crystal upon DSC heating, leading to nonisothermal cold crystallization at a much lower temperature than that of the melt-quenched sample. For the same *t*_t_, *T*_c_ decreases with increasing CO_2_ pressure because of the much higher degree of order. As shown in the inset of Figure 8b, the difference (Δ*T*_c_) in *T*_c_ between the melt-quenched and CO_2_-treated samples approaches a constant value in a very short *t*_t_ of 10^1^ min for 2.0 MPa, whereas Δ*T*_c_ for 1.8 MPa starts to increase at the same *t*_t_. Nonetheless, the maximum Δ*T*_c_ for samples treated at 1.8 MPa is still lower than that of those at 2.0 MPa. For the case of 1.6 MPa, Δ*T*_c_ only slightly increases with increasing *t*_t_ and is considerably smaller than those of 1.8 MPa and 2.0 MPa. However, it should be stressed that *T*_c_ is significantly lower for all CO_2_-treated samples than for the melt-quenched sample. That is, as shown in the figure, Δ*T*_c_ ≥ ca. 15 °C for all treated samples, indicating a significant degree of order in the formed mesophase. Nonetheless, in terms of Δ*T*_c_, the mesophase formed at 1.6 MPa has a much lower degree of order when compared to those at higher CO_2_ pressures, which agrees well with the IR results discussed in Figure 2 and Figure 3. Therefore, the thermal behavior from the DSC results demonstrates that CO_2_ pressure dominates both the scale and dynamics of chain motion, which further affects the kinetics of mesophase formation and the resulting degree of order.

At a constant temperature, the solubility of CO_2_ in polymers generally increases with increasing pressure. According to the literature, it is known that the solubility of CO_2_ in polylactide increases with increasing pressure at 0 °C [49]. In particular, there are favorable intermolecular forces between CO_2_ molecules and PLLA chains, which results in a high degree of solubility at low temperatures. The solubilities of CO_2_ in PLLA at 2.0, 1.8, and 1.6 MPa are approximately 15, 13.5, and 12 wt.% [49], respectively. In particular, higher pressures lead to an increased free volume fraction, consequently enhancing the mobility of PLLA molecular chains to a greater extent. Specifically, the increase in molecular chain mobility involves the scale and dynamics of chain motion. Compared to mesophase formation under atmospheric pressure, PLLA chains under low-pressure CO_2_ conditions are more likely to undergo *gg*-*gt* conformational changes to a limited extent and promote mesophase formation, which helps the system to search for a relatively lower Gibbs free energy. The lower pressure (1.6 MPa) and the consequently low CO_2_ solubility lead to slower dynamics and a smaller scale of chain motion, resulting in slower *gg*-*gt* conformational changes and a lower degree of order in the mesophase. Moreover, the mesophase has a degree of order far from that of a crystalline phase, in particular for that formed at 1.6 MPa. The scale and dynamics of chain motion under lower pressure (1.6 MPa) are significantly smaller than those of cooperative segmental mobility and do not disrupt the aggregated state formed by enthalpy relaxation (physical aging) prior to CO_2_ treatment.

The multistage model of crystallization suggests that the growth of the lamellar crystallites is a multistep process that passes through intermediate states [25]. The present work demonstrates that different chain mobility, which is within a suitable range (otherwise crystallization occurs), promotes the formation of the intermediate phase (mesophase) with distinct kinetics and varying degrees of order. This may account for the role of a higher PEG concentration in enhancing the crystal growth rate of PLLA [50]. The presence of more PEG likely promotes the formation of more mesophase on the growth front owing to enhanced chain mobility. The present findings may also help to explain the very limited mesophase formation due to physical aging [19], in which both the scale and dynamics of chain motion at temperatures well below the *T*_g_ are remarkably limited.

## 4. Conclusions

Using IR spectroscopy, DSC, and AFM, the impact of varying chain mobility on the dynamics of mesophase formation in melt-quenched PLLA was investigated by adjusting CO_2_ pressure and hence the CO_2_ solubility under mobility-limited conditions under low-pressure (1.6–2.0 MPa) CO_2_ at 0 °C. The IR results showed that the final degree of order and kinetics of structural evolution during the CO_2_-induced mesophase formation were dependent on the CO_2_ pressure. When compared to the untreated sample, the enhancements in the final degree of order were approximately 10, 19, and 27% for treatments at 1.6, 1.8, and 2.0 MPa, respectively. This was attributed to the distinct dynamics of conformational evolution (*gg* to *gt* conformer transition) due to the different CO_2_ pressures. AFM morphology results also supported that the CO_2_ treatment pressure impacted the formation process of the mesophase. On the basis of the DSC results, the thermal behavior indicated that CO_2_ pressure not only influenced the scale but also the dynamics of the chain motion of PLLA chains. At a lower CO_2_ pressure of 1.6 MPa, smaller-scale segmental motion could not be replaced by the larger-scale cooperative motion occurred at the relatively higher CO_2_ pressure of 2 MPa, which was favorable for faster mesophase formation. Despite the mesophase formation to some extent under the lower CO_2_ pressure of 1.6 MPa, the aggregated state formed by previous enthalpy relaxation prior to CO_2_ treatment had not been destroyed. Consequently, by inhibiting direct crystallization under limited mobility conditions, it was demonstrated that varying chain mobility controlled by CO_2_ solubility impacted the dynamics of the mesophase formation of PLLA. The present results have implications for understanding the role of chain mobility in determining the intermediate structural phases in semicrystalline polymers including PLLA.

## Data Availability

Data is contained within the article.

## Supplementary Materials

The following supporting information can be downloaded at: https://www.mdpi.com/article/10.3390/polym16101378/s1, Figure S1: FTIR spectra with full range for samples corresponding to Figure 1; Figure S2: DSC curve of melt-quenched sample.
