# Supplementary material for: Understanding the Impact of Chain Mobility on Conformational Evolution and Kinetics of Mesophase Formation in Poly(ʟ-lactide) under Low-Pressure CO2
Source: Polymers (Basel). 2024 May 12;16(10):1378. doi: 10.3390/polym16101378 (PMC11124961; doi:10.3390/polym16101378)
Supplement: Supplementary file 1 [file polymers-16-01378-s001.zip › polymers-2999580-supplementary.pdf]

Supplementary Materials

Figures S1–S2.

Understanding the Impact of Chain Mobility on Conformational Evolution and Kinetics of Mesophase Formation in Poly(L-lactide) under Low-Pressure CO_2

Youjuan Liao and Qiaofeng Lan *

Biomaterials Research Center, School of Biomedical Engineering, Southern Medical University, Guangzhou 510515, China; liaoyjcn@126.com
* Correspondence: lanqf@smu.edu.cn

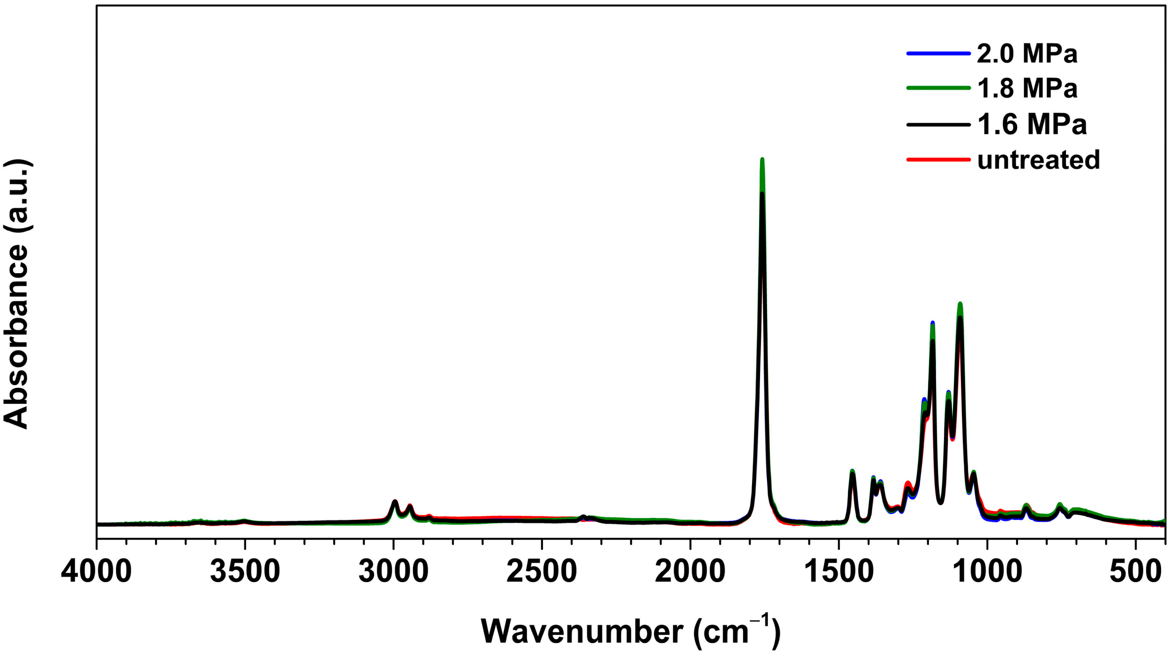

Figure S1. FTIR spectra in the wavenumber ranges of 4000–400 cm^{-1} for PLLA films treated under CO2 at 1.6–2.0 MPa and 0 °C for 12 h. The spectrum of melt-quenched/untreated sample is also shown for comparison.

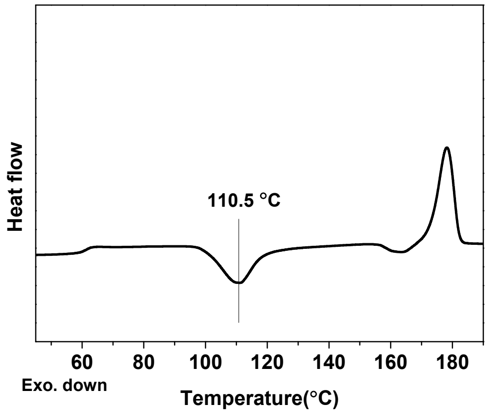

Figure S2. DSC curve (second heating) of melt-quenched (cooling at a rate of 40 °C/min after melting at 210 °C) PLLA sample.
